# Effect of non-surgical periodontal therapy on serum ferritin levels in postmenopausal women with chronic periodontitis

**DOI:** 10.34172/joddd.2021.030

**Published:** 2021-08-25

**Authors:** Masoumeh Faramarzi, Adileh Shirmohammadi, Azin Khorramdel, Mehrnoosh Sadighi, Elshan Bargahi

**Affiliations:** ^1^Department of Periodontics, Faculty of Dentistry, Tabriz University of Medical Science, Tabriz, Iran; ^2^Private Practitioner, Tabriz, Iran

**Keywords:** Chronic periodontitis, Ferritin, Menopause, Non-surgical periodontal treatment

## Abstract

**Background.** Ferritin is a positive acute phase protein (APP) in inflammation and chronic infections, including chronic periodontitis. Two key factors that can regulate ferritin expression are iron and pro-inflammatory cytokines. Serum ferritin levels increase after menopause, affecting women’s health. This study aimed to evaluate serum ferritin levels in postmenopausal women upon undertaking non-surgical periodontal treatment.

**Methods.** In this cross-sectional study, blood samples of 38 postmenopausal women with chronic periodontitis were collected before any treatment. The serum ferritin levels and periodontal parameters, probing depth (PD), clinical attachment level (CAL), and gingival index (GI) were recorded at baseline and three months after non-surgical periodontal therapy. Wilcoxon test was used to compare serum ferritin levels before and after treatment. T-test was used for comparison of periodontal parameters, with a *P* value of ≤0.05 considered significant.

**Results.** A decrease was observed in the serum ferritin level (from 108.55 mcg/L to 98.28 mcg/L) after treatment compared to baseline (*P* < 0.001). Also, significant improvements in periodontal parameters were observed compared to the baseline (*P* < 0.001).

**Conclusion.** Based on the results, it can be concluded that non-surgical periodontal treatment significantly reduces serum ferritin levels in postmenopausal women with chronic periodontitis.

## Introduction

Periodontal disease is an inflammatory condition of tooth-supporting structures caused by the subgingival accumulation of anaerobic gram-negative bacteria and characterized by progressive destruction of periodontal tissues.^1^

Periodontitis is a prevalent disease worldwide and is a significant health problem in many countries.^2,3^ Early diagnosis and treatment of this disease prevents its progression.^4^

Nowadays, the diagnosis of periodontitis focuses on clinical measurements, i.e., probing depth (PD), clinical attachment level (CAL), bleeding on probing (BOP), and radiographic findings.^5^ These parameters often indicate previous periodontal disease rather than the current activity of the disease. Therefore, new diagnostic tests are necessary to determine the presence of disease activity, its future progression, and evaluation of the response to periodontal treatment due to clinical improvements in periodontitis patients.

Recent research on periodontal disease diagnosis focuses on how periodontitis risk can be identified by objective measurements such as biomarkers. Biomarkers are molecules that can be used to monitor health, disease onset, response to treatment, and treatment outcomes, including biomarkers of acute phase proteins (APPs).^6^

Changes in the concentration of APPs are a physiological phenomenon resulting from inflammation and tissue damage. APPs can be associated with a wide range of disorders, including infection, trauma, infarction, inflammation, and various neoplasms.^7,8^

Ferritin is an acute-phase reactant that increases during inflammation, autoimmune disease, chronic infection, and liver disease. Elevated ferritin serum levels have been reported in many chronic inflammations associated with conditions such as adult-onset Still’s disease, MS, and rheumatoid arthritis.^9,10^ In addition to its role as an APP, ferritin assumes an essential role in iron storage. Ferritin stores iron in a non-toxic, soluble form and releases it in a controlled manner.^11^

Ferritin also has a vital role in the host’s immune response. As the body’s immune response increases, so does the migration of ferritin from the plasma to the cells as a means of countering pathogens. This is an attempt to bind iron with the host tissue.^12^ Two key factors that control ferritin expression are iron^13^ and the pre-inflammatory factors cytokines.^14,15^ Both estrogen and iron are the most crucial growth nutrients in female body development. Lack of estrogen in postmenopausal women is considered the leading cause of menopausal symptoms and diseases due to estrogen affecting the growth, differentiation, and functionality of tissues, such as breasts, skin, and bones.^16^ Iron is also essential for oxygen transport, DNA synthesis, and energy production.^17^

During postmenopausal changes, estrogen levels decrease due to ovarian failure, but iron levels increase due to the cessation of menstrual periods.^18,19^ These observations could form a hypothesis that in addition to reducing estrogen, an increase in iron levels could be a risk factor for postmenopausal women’s health.^16^

Elevated iron levels lead to increased proliferation of osteoblastic progenitors without their differentiation into adult osteoblasts. Therefore, bone formation becomes slower, and the increased iron leads to oxidative stress and skin sensitization to ultraviolet exposure.^16^ Also, the high prevalence of heart disease in postmenopausal women and men can be attributed to the high levels of iron in these two groups.^20^ The differences between postmenopausal women and men in their hormones and iron levels cannot be neglected. Increased iron in men is associated with an increase in male sex hormones, which can lead to protection against the harmful effects of iron.^18,21^

On the other hand, it has been shown that ferritin increases as a positive APP in inflammation and chronic infection, including chronic periodontitis, decreasing the serum levels of this index following periodontal treatment.^22^

Considering the side effects of increased ferritin levels in postmenopausal women and the impact of periodontal infection on increasing ferritin levels, this study aimed to evaluate the changes in serum ferritin levels in postmenopausal women following non-surgical periodontal disease treatment.

## Methods

This cross-sectional study was performed on postmenopausal women referring to the Department of Periodontics, Faculty of Dentistry, Tabriz University of Medical Sciences. Written consent was obtained from all the subjects enrolled in the study.

Inclusion criteria were as follows: non-smoking postmenopausal women with systemic health, > 50 years of age, with at least one year since the onset of their menopause; patients with chronic periodontitis with at least 20 teeth with BOP and attachment loss of 3-4 mm or a PD of ≥5.

Exclusion criteria were as follows: a history of taking non-steroidal anti-inflammatory drugs (NSAIDs) and antimicrobial drug in the last six months before the study; a history of any periodontal treatment in the six months before the survey; the presence of any systemic disease (such as iron deficiency anemia, hemochromatosis, diabetes, etc.) and infectious conditions other than chronic periodontitis; the presence of aggressive periodontitis, use of mouthwashes and vitamin supplements in the last three months before the study.

Thirty-eight postmenopausal women were selected, with generalized moderate chronic periodontitis (attachment loss of 3‒4 mm and radiographic evidence of bone loss in more than 30% of the areas) in at least 20 teeth. Blood samples were collected from all participants at baseline and after three months by professional operators. The blood samples were transferred into sterile vacuum tubes with no anticoagulants and sent to the laboratory in less than two hours. An automated analyzer (Tosoh Co., Japan) was used to measure ferritin serum levels using an enzymatic immunoassay technique. Then, the clinical parameters, such as PD, CAL in six areas of each tooth (mesiobuccal, buccal, distobuccal, distolingual, lingual, and mesiolingual), BOP (BOP),^23^ gingival index (GI),^24^ and plaque index (PI),^25^ were measured in patients with chronic periodontitis at baseline using a mirror and a UNC-15 periodontal probe (UNC-15, Hu-Friedy, Chicago, IL, USA ). One examiner performed all the examinations to prevent interexaminer errors.

Afterward, non-surgical periodontal treatments, including oral hygiene instruction, scaling and root planing, and a two-week oral administration of 0.12% chlorhexidine, were performed for chronic periodontitis patients. After the treatment and a follow-up period of three months, venous blood samples were collected again to determine serum ferritin levels, followed by the re-assessment of clinical parameters.

The data were reported using descriptive statistical methods (mean ± SD). The normal distribution of variables was verified using the Kolmogorov-Smirnov test. To compare ferritin levels before and after non-surgical treatment after confirming the normal distribution of data, paired t-test was used. In the case of non-normality of data, the nonparametric equivalent, i.e., the Wilcoxon test, was applied. In this study, *P*≤0.05 was considered statistically significant. All the analyses were performed using SPSS 17 (SPSS Inc., IL, Chicago, USA).

## Results

Statistical evaluation of clinical and biochemical parameters yielded the following results. Table 1 shows the means and standard deviations of serum ferritin levels, PD, CAL, BOP, GI, and PI in postmenopausal women with chronic periodontitis before non-surgical treatment and three months after treatment.

**Table 1:** Descriptive clinical and biochemical parameters of the subjects at baseline and 3 month post treatment

**Parameters**	**Mean±SD**		***P*** ** value**
	**Baseline**	**3 month post treatment**	
Serum ferritin levels (mcg / L)	108.55±26.08	98.28±23.65	0.001
PD (mm)	5.27±0.53	3.92±0.62	0.001
CAL (mm)	3.98±0.56	2.64±0.47	0.001
BOP (%)	53.49±14.3	10.41±3.45	0.001
GI	0.92±0.81	0.63±0.24	0.001
PI	70.02±11.93	23.71±3.36	0.001

The serum ferritin levels of postmenopausal women with chronic periodontitis before treatment was 108.55 mcg/L, which decreased to 98.28 mcg/L after treatment. Other variables such as PD decreased from 5.27 mm to 3.92 mm after treatment; CAL decreased from 3.98 mm to 2.64 mm, BOP from 53.49 to 10.41, GI from 0.92 to 63.62, and PI from 70.02% to 23.71%. Thus, all of the periodontal parameters decreased after treatment.

To select appropriate statistical tests to analyze the collected data, it is necessary to evaluate the distribution of variables in terms of their normal distribution, in which the Kolmogorov-Smirnov test was used. It was observed that the distribution of serum ferritin levels before treatment was not normal because its value was less than 0.05. Therefore, a nonparametric test, such as Wilcoxon’s test, was used to compare the serum ferritin levels before and after treatment. For other variables, the t-test was used due to their normal distribution. In all of the parameters, a statistically significant decrease was observed from the baseline to 3 months after treatment (*P*≤0.05) (Table 1).

## Discussion

Periodontitis is an inflammatory disease of tooth-supporting tissues caused by subgingival anaerobic bacteria.^26^ Serum levels of APPs change in patients with chronic periodontitis. APPs are those whose concentrations change at least up to 25% when responding to inflammation, including ferritin and transferrin. Ferritin is an essential store of iron in the body with an average level of 18‒160 mcg/L in females.^27,28^

High levels of ferritin in women indicate impaired iron storage and chronic inflammatory conditions, such as liver disease, rheumatoid arthritis, and some cancers; however, low ferritin levels are observed in iron deficiency. High levels of ferritin have also been reported in chronic periodontitis.^29^

In the present study, the effect of non-surgical periodontal treatment on the serum ferritin levels in postmenopausal women was investigated. The results showed that the mean serum ferritin level in postmenopausal women with chronic periodontitis at the beginning of treatment was 108.58 mc/L, which decreased to 98.28 mcg/L after treatment. Chakraborty et al^22^ found that serum ferritin levels were higher in patients with chronic periodontitis, which decreased after periodontal treatment, consistent with the present study. In addition, it has been reported that elevated serum ferritin levels might be associated with the severity of periodontitis.^30^ However, in a survey by Latha et al,^29^ there was no difference in serum ferritin levels between the periodontitis group and the control group, which might be due to differences in race, sample size, and input standards.

Previous studies showed that after non-surgical periodontal treatment, ferritin levels decreased in patients with chronic periodontitis, indicating that iron overload and inflammation rate improved after treatment.^22,31^ The present study provided evidence on the importance of periodontitis control with periodontal treatment. The key findings of this study were that a decrease in periodontal inflammation with non-surgical treatment reduces serum ferritin levels compared to baseline. After non-surgical periodontal treatment, changes in serum ferritin levels were associated with improved periodontal pocket depths. Three months after periodontal treatment, all the periodontal parameters improved along with lower serum ferritin levels. Non-surgical periodontal treatment improved periodontal inflammation, decreasing serum ferritin levels in patients with periodontitis. The findings of the present study also revealed that the PD at baseline, which was 5.27 mm, decreased to 3.92 mm three months after treatment; CAL, which was 3.88 mm at the baseline, decreased to 2.64 mm at the end of the third month. Three other variables decreased after treatment; BOP decreased from 53.49 to 10.41, GI from 0.92 to 0.63, and PI from 70.02% to 23.71%.

A study by Daltaban et al^32^ on the effects of periodontal treatment in postmenopausal women showed that PI, GI, BOP, PD, and CAL in patients with periodontitis were significantly higher than the control group. Also, in the present study, these variables decreased after treatment, consistent with the study above.

Badersten et al^33^ examined the effects of non-surgical periodontal therapy in 16 patients with periodontitis, including 11 men and 5 women, with an age range of 38 to 58 years. In each patient, 4 to 10 teeth were examined. Measurements of PI, GI, BOP, and PD were performed three months after treatment. The results showed that all the parameters decreased after treatment.

## Conclusion

According to the present study and its comparison with other studies, in general, non-surgical periodontal treatment significantly reduces serum ferritin levels, PI, GI, BOP, PD, and CAL in postmenopausal women with chronic periodontitis.

## Authors’ contributions

EB and AS performed the initial examination and patient assessment and reviewed the available literature. MF carried out periodontal treatment. MS and AK prepared the manuscript. All the authors have read and approved the final manuscript.

## Acknowledgments

The authors would like to acknowledge the Dental and Periodontal Research Center, Tabriz University of Medical Sciences, for financial support of this project.

## Funding

The study was supported by the Dental and Periodontal Research Center, Faculty of Dentistry, Tabriz University of Medical Sciences.

## Competing interests

The authors declare that they have no competing interests regarding authorship and/or publications of this paper.

## Ethics approval

The study protocol was approved by the Ethics Committee of Tabriz University of Medical Sciences under the code TBZMED.REC.1397.1031
